# Association between long-term, short-term spatial land surface temperature variations and health-related quality of life in Lima, Peru

**DOI:** 10.1186/s12942-026-00454-w

**Published:** 2026-02-13

**Authors:** Antonio Torres-Reyes, Pablo S C Santos, Yanetsy Elisa Rodríguez León, Estefanie Quispe Salas, Alessandra Gudila Rodriguez Mercado, Melvine Anyango Otieno, Elvis Ndikum Achiri, Jan W. Kantelhardt, Andreas Wienke, Jan Christian Schlüter, Eva Johanna Kantelhardt

**Affiliations:** 1 Global and Planetary Health Working Group, Institute of Medical Epidemiology, Biometrics and Informatics, Martin-Luther-University Halle-Wittenberg, Halle (Saale), Germany; 2 Chair of Data Science, Faculty of Management, Construction and Real State, HAWK, Holzminden, Germany; 3 Department of Applied Mathematics, Faculty of Mathematics and Computer Sciences, University of Havana, Havana, Cuba; 4 University of Lima, Lima, Perú; 5 Department of Urban Planning and Design Harvard Graduate School of Design, Harvard University, Cambridge, MA USA; 6 Institute of Physics, Martin-Luther-University, Halle (Saale), Germany; 7 Institute of Medical Epidemiology, Biometrics and Informatics, Martin-Luther- University Halle-Wittenberg, Halle (Saale), Germany; 8 Computational Epidemiology and Public Health Research Group, Institute for Medical Epidemiology, Biometrics and Informatics, Interdisciplinary Center for Health Sciences, Martin Luther University Halle-Wittenberg, Halle (Saale), Germany; 9 Department of Gynecology, Martin Luther University Halle-Wittenberg, Halle (Saale), Germany

**Keywords:** LST, Self-rated health, Ageing, HRQOL, EQ-5D-5L cross-sectional, Lima

## Abstract

**Background:**

The effect of environmental exposures on self-reported health has gained attention in the literature in recent years. Rising temperatures have become a public health concern due to climate change and urbanisation at a global scale. Findings vary strongly across regions, highlighting the need for further evidence to inform data-driven decisions.

**Methods:**

This study aimed to assess the impact of land surface temperature (LST) on health-related quality of life (HRQOL) among adults in Lima, Peru. We assessed 700 randomly selected adults using structured interviews covering HRQOL, sociodemographic factors, behavioural habits, and non-communicable disease (NCD) prevalence, between December 2023 and January 2024. LST was derived from satellite-based data.

**Results:**

We detected no association between LST and self-reported health for most of the exposure timeframes. However, we observed an association between spring and summer mean temperature for reporting perfect health and reporting problems in the usual activity and anxiety or depression dimension, respectively.

**Conclusions:**

These findings demonstrate the complex interplay of LST as an environmental exposure and HRQOL in urban settings. Insights from this study can optimise public health policies and interventions aimed at promoting healthy behaviours, improving environmental conditions, and enhancing population well-being. Further research should investigate the observed associations across diverse geographic settings and develop targeted strategies to improve HRQOL in the context of rising global temperatures.

**Supplementary Information:**

The online version contains supplementary material available at 10.1186/s12942-026-00454-w.

## Background

Environmental epidemiology assesses the interplay between environmental exposures and health outcomes [1]. According to estimates from 2016, 24.3% of deaths globally were linked to environmental factors including heat and air pollution [2]. Temperature-related mortality is a pivotal concern due to climate change and increasing global temperatures. An estimated 5.08 million deaths in 2021 (9.4% of all deaths globally) were attributed, solely, to non-optimal temperatures [3]. In Latin America, a study examining deaths between 2002 and 2015 estimated that 5.75% of all deaths were associated with non-optimal temperatures [4].

High and low ambient temperatures have been associated with various adverse health outcomes, including increased infant and elderly mortality, hospital admissions, and respiratory diseases [5–7]. Rising global temperatures, largely due to climate change, have intensified health risks, contributing to non-communicable diseases (NCDs), infectious vector-borne diseases, malnutrition, and increased mortality during high heat events [8, 9].

Nevertheless, the impact of temperature on broader health-related quality of life (HRQOL) remains less understood [10]. HRQOL encompasses a holistic view of well-being, aligned with the World Health Organization’s (WHO’s) definition of health as “*a state of complete physical*,* mental*,* and social well-being*,* not merely the absence of disease or infirmity*” [11]. This concept has been operationalised through tools such as the EQ-5D-5L questionnaire, which evaluates HRQOL across five dimensions: mobility, self-care, usual activities, pain or discomfort, and anxiety or depression, each rated on a five-level severity scale [12, 13]. The EQ-5D-5L has been employed in the Hispanic American context and it is validated for use in Peru [14, 15].

Although some studies have examined the influence of temperature on well-being, the findings have been inconsistent. For instance, a study in Japan among university students suggested optimal well-being at a mean temperature of 17.5 °C, with extremely hot temperatures detrimentally affecting emotional well-being [16]. A study in Southern Germany found no association between average short-term daily temperature and HRQOL, measured with the EQ-5D-5L instrument, but observed a protective effect of moderate and extreme cold on the usual activity dimension [17]. In Australia, both cold and hot temperatures were reported to negatively impact health [18]. In several Latin American cities, elevated temperatures were shown to be associated with an increased mortality risk [19].

Individual factors like age and sex [19], as well as socio-economic factors such as education, income or living conditions may buffer some temperature-related risks [20–23]. Oraiopoulos et al. reported indoor temperatures outside the comfort zone in an informal settlement in Lima, although householders frequently reported satisfaction with their home’s thermal conditions, highlighting the complexity of humans’ perception of heat [24]. But previous health conditions like non-communicable diseases (NCDs) may mediate temperature-related risks and individual responses [25]. The influence of temperature on HRQOL remains largely underexplored, especially in rapidly urbanising regions of the Global South, where environmental and infrastructural challenges may exacerbate these this potential influence.

This study focused on Lima, Peru, a city facing rapid urbanisation, high temperatures [26], significant air pollution [27], and limited infrastructure [28, 29]. These conditions match many cities in the Global South, providing an opportunity to investigate the influence of environmental factors on HRQOL within urban populations.

Four environmental parameters influence body heat storage: ambient temperature, absolute humidity, air velocity, and mean radiant temperature (MRT) [30]​. This study utilizes Land Surface Temperature (LST) as a proxy for MRT, acknowledging that these metrics capture different aspects of urban thermal conditions. LST, often derived from satellite data, typically reflects Urban Heat Island (UHI) patterns, driven primarily by two-dimensional surface properties such as albedo and land cover classes. MRT, conversely, is a composite indicator that characterizes the thermal burden on the human body at hyper-local scales (teens of meters), responding significantly to three-dimensional urban design features like shading and building height [31].

Although MRT is a more comprehensive measure for quantifying human heat exposure, LST remains the most widely adopted metric because its maps are readily available from free remote sensing products over large spatial extents, whereas high-resolution MRT requires complex microclimate modelling and intensive field data collection [32]. Despite LST being an imperfect proxy for thermal comfort, previous analysis revealed that remotely sensed LST explained approximately 40% of the variance observed in simulated MRT during the daytime [31] and it has been reported to be relevant for mortality [33] and morbidity [34].

While mortality and morbidity have traditionally been the primary health outcomes in environmental health studies [35, 36], understanding the potential effect of temperature on HRQOL can provide additional insights into overall well-being. From a regional climatic perspective, most of the evidence has been reported for the Global North [17, 37, 38], while more research is needed for the Global South. To our knowledge, this is the first study to assess the impact of LST on HRQOL in a low and middle-income country using remote sensing data. Such evidence can inform comprehensive policies aimed at improving population health and adapting to the challenges posed by climate change.

### Objective

This study aimed at assessing possible long- and short-term associations between temperature (measured as LST) and quality of life (measured as HRQOL) among adults in Lima, Peru. Specifically, we investigated the influence of environmental factors on the likelihood of participants to report HRQOL problems across various dimensions.

## Methodology

This study employed a cross-sectional, household-based data collection approach in Lima, Peru. Lima is the country’s largest city and the capital, with an estimated population of 10.3 million [39], 28.7% of whom live in unplanned urban areas [40]. Lima’s climate is classified as a hot desert [41] under the Köppen-Geiger system, with a mean annual temperature of 19.4 °C and 86% humidity [42, 43].

### Study participants and sampling strategy

Adults aged 18 or above who had resided at their current address for at least one year, spoke Spanish, had no physical or cognitive impairments that could interfere with the interview process and were willing to participate in the study were deemed eligible for this study. We applied a spatial sampling approach, assigning 800 target households. However, after data cleaning, 700 were employed in the data analysis. Using the “sample points” function from the Geopandas Python library, random points were generated across residential blocks proportional to the number of houses per block. Surveyors visited these points and interviewed residents from selected households, moving to the nearest available home if residents were absent. Data collection occurred over two periods: November 25 to December 16, 2023, and January 17 to January 27, 2024. The surveyors initially recorded the data on paper and digitised the data later. GPS coordinates and timestamps were collected using KoBoToolbox [44].

### Health and sociodemographic data collection

The EQ-5D-5L questionnaire, in self-administered form, was used to measure HRQOL across five dimensions: mobility, self-care, usual activities, pain or discomfort, and anxiety ordepression. The responses were rated on a five-level Likert scale. The EQ-5D-5L utility score ranged from 0 (death) to 1 (perfect health). The participants’ health behaviours were documented using the WHO STEPwise approach [45], with minor adaptations for the local context. Non-communicable disease diagnoses were self-reported from a list provided by the surveyors. Questions on housing materials (floor and wall types) were included to account for thermal insulation effects. Education level and family mean income were collected using the categories employed in ENDES, the Peruvian National Household Survey. We also collected the age of the participants.

### Environmental exposure measurement

Temperature and air pollution data were sourced from satellite products to ensure cost-effectiveness and enough spatial resolution to compare different points within the city. The LST data was obtained from the Landsat [46] Collection 2, with 30 m spatial resolution and a revisit time of 16 days. The statistical mono-window (SMW) algorithm, as implemented in Google Earth Engine, was used to convert the top-of-atmosphere brightness temperature to LST [47]. LST were measured in a radius of 30 m around the house location. We constructed a 10-year time series, 01.2014 to 01.2024 for each household and mapped the measurements to a single fictitious year. Then, we applied a cosine function to model seasonal variation and avoid time-sampling inconsistencies. This approach yielded a time series and seasonal mean values for robust estimates and allows us to explore the long-term association. To explore the short-term, we used the LST measurement closer to the interview date.

### Potential confounders and biases

We collected potential covariates following literature reports and operationalized the following as categorical variables [48]: sex, education, monthly income, house floor and wall materials and smoking habits. On the other hand, age, NCDs incidence, net vegetable and fruit intake per week and net hours spent weekly walking or biking were collected as continuous variables. A stepwise backward selection method minimizing the Bayesian Information Criterion (BIC) was used to select from the initial set of covariates. Although this procedure would have excluded a potential covariate (as happened with age) identified in the literature, we retained it in the final set due to its relevance to the study topic. The final model only included age, sex, NCDs incidence, education and monthly income.

Age was included as both a continuous and categorical variable in descriptive analyses but modelled as continuous in association analyses. Sex was coded as a binary variable, “male” and “female”. The incidence of non-communicable diseases (NCDs) was calculated as the total number of reported NCDs. Education was treated as a categorical variable with five levels: “No education”, “Primary completed”, “Secondary completed,” “Technical education,” and “University or postgraduate”. For the regression analysis, this variable was employed as ordinal with “No education” being the button category and “University or postgraduate” the top category. Income was originally collected in Peruvian soles (later converted to USD) across five categories: “<342”, “343–653”, “654–998”, “999–1850”, and “>1851” and subsequently reencoded as an ordinal variable ranging from 1 (“<342”) to 5 (“>1851”). To mitigate information bias, we used standard questionnaires to collect the data. The spatial random sampling approach decreased the chance of selection bias. We followed the **STR**engthening the Reporting of **OB**servational studies in **E**pidemiology (STROBE) checklist for research conception and reporting results [49].

### Statistical analysis

The EQ-5D-5L index was computed using the EQ-5D R package [50], with the value set for Lima derived from a discrete choice experiment [14]. We dichotomized the EQ-5D-5L dimensions as follows: “no problems in” when a participant reported perfect health (level 1) and “some problem” when a health problem was reported (level 2 to 5). The obtained binary variable helped to investigate the effect of covariates on the likelihood of reporting a health problem, irrespective of the severity of the problem. Missing values were handled by excluding records with missing temperatures (*n* = 4) and income (*n* = 96). Given the potential bias and uncertainty in self-reported income data, we chose not to impute the 96 missing values to prevent adding further noise to the dataset. Spatial autocorrelation of the dependent variable was tested using Moran’s I (Moran, 1950), and no autocorrelation was found (Figure S1 in supplementary materials). We also tested for correlations among the covariates, and non-strong correlations were observed (Table S1 in supplementary materials).

We used a beta one-inflated (BEOI) regression model to account for the distribution of the EQ-5D-5L utility index, which ranged between 0 and 1 with a high proportion of exact ones (37.1% in the sample). The BEOI model jointly estimates two components: (1) a beta regression for values strictly between 0 and 1, and (2) a logistic model for the probability of observing an outcome equal to one [51]. Multivariable logistic regressions were conducted for each EQ-5D-5L dimension as a dependent variable using the dichotomised variables.

Six temperature exposure metrics were analysed: for capturing the long-term exposure the 10-year mean LST, seasonal mean LSTs for spring, summer, autumn, and winter were employed. The LST closest to the interview date was used to capture the short-term exposure. Each metric was entered independently into the models to isolate its association with HRQOL outcomes. Separate models were fitted for each temperature variable using both the BEOI regression for the EQ-5D-5L utility index and multivariable logistic regressions for the EQ-5D-5L dimensions. Their results were presented as odds ratios with 95% confidence intervals.

### Sensitivity analyses

To evaluate the robustness of our findings, we conducted several sensitivity analyses. We stratified the models by sex and income level (low: ≤653 $; high: ≥654 $), comparing coefficient magnitudes and directionality across groups. We run the model for female population and low income level, for the high income level, the model did not converge probably due to small sample size, *n* = 101. Later we tested potential interactions between temperature exposure and both age and income in the main models; none reached statistical significance (*p* > 0.1). The results of this analysis are reported in full in the supplementary materials: SM_LogReg_Sensitivity and SM_BEOI_Sensitivity.

We compared the LST values to air temperature (Ta) measurements from the nearest ground-based weather stations across different seasons and locations. Observations extended from January 2019 to October 2024, having four years of overlap with LST measurements. Data processing, analysis and visualisations were performed using Python and R, and the code is accessible at https://bunsencloud.de/s/H9NSYGnTYgsf2EL.

### Ethical considerations

This study was carried out following the principles of the Declaration of Helsinki. The study also received ethical approval from the institutional review board of the Medical Faculty of Martin Luther University Halle-Wittenberg on November 2, 2023 (processing number 2023 − 219) and from the ethics board of Universidad Católica del Perú on November 23, 2023 (resolution number 141–2023-CEI-CCSSHHyAA/PUCP). Every participants provided consent to participate in the study.

## Results

Table 1 presents the sociodemographic characteristics of the sample. The age distribution of the participants closely resembled that of the Peruvian population [39]. Overall, 62.9% of the participants reported HRQOL problems. Of the female participants, 69.7% manifested some HRQOL problems, while 53.3% of the men did.

The EQ-5D-5L utility score averaged 0.91 (SD = 0.11) across the sample, ranging from 0.2 to 1.0. This score was higher among men than women, increased with higher education and income levels. Older-aged groups reported lower HRQOL than younger-aged groups. The 18–29 age group had the highest average utility score (0.96, SD = 0.06), while those aged 70 + had the lowest (0.84, SD = 0.16).

Other sociodemographic factors showed similar trends. The participants with university or postgraduate education reported higher HRQOL (mean utility score of 0.95, SD = 0.06) than those with no formal education (mean = 0.84, SD = 0.14). Similarly, the participants with monthly incomes exceeding USD 1851 had the highest HRQOL scores (mean = 0.96, SD = 0.05), while those earning below USD 342 had the lowest scores (mean = 0.90, SD = 0.12). These descriptive statistics suggest that sociodemographic variables such as age, sex, education, and income may be relevant in explaining HRQOL.

**Table 1:** Descriptive statistics of the sample *n* = 700, total, segregated by reporting or not health-related problems and utility index

	Categories	Total N=700 **n(%)**	No health problem, ** n (%)** 260 (37.1)	Some health problem, **n (%)** 440 (62.9)	EQ-5D-5L index **mean (SD)** 0.91, (0.11)
Age groups, years	18-29	128 (18.3)	68 (26.2)	60 (13.6)	0.96 (0.06)
	30-49	315 (45.0)	129 (49.6)	186 (42.3)	0.93 (0.08)
	50-69	198 (28.3)	51 (19.6)	147 (33.4)	0.88 (0.13)
	70+	59 (8.4)	12 (4.6)	47 (10.7)	0.84 (0.16)
Sex Group	Female	409 (58.4)	124 (47.7)	285 (64.8)	0.90 (0.12)
	Male	291 (41.6)	136 (52.3)	155 (35.2)	0.93 (0.10)
Education levels	No education	48 (6.9)	8 (3.1)	40 (9.1)	0.84 (0.14)
	Primary completed	125 (17.9)	32 (12.3)	93 (21.1)	0.87 (0.14)
	Secondary completed	493 (62.7)	182 (70.0)	257 (58.4)	0.93 (0.10)
	Technical education	48 (6.9)	22 (8.5)	26 (5.9)	0.94 (0.08)
	University or postgraduates	40 (5.7)	16 (6.2)	24 (5.5)	0.95 (0.06)
Income levels(USD*)	< 342	380 (54.3)	124 (47.7)	256 (58.2)	0.90 (0.12)
	343-653	219 (31.3)	88 (33.8)	131 (29.8)	0.92 (0.11)
	654-998	55 (7.9)	25 (9.6)	30 (6.8)	0.94 (0.09)
	999-1850	32 (4.6)	15 (5.8)	17 (3.9)	0.95 (0.06)
	>1851	14 (2.0)	8 (3.1)	6 (1.4)	0.96 (0.05)

		**mean(SD)**	**mean(SD)**	**mean(SD)**	
Age, mean(SD)		44.9 (15.8)	39.9 (14.3)	47.8 (15.9)	
NCDs count, mean(SD)		0.6 (0.8)	0.3 (0.5)	0.8 (0.9)	

*Peruvian Sol to USD exchange rate from 06.09.2024

The overall mean LST (Table 2) was 26.4 °C (SD = 2.8), ranging from 20.2 to 33.0 °C. Participants reporting some health problems were exposed to slightly higher average LST (26.5 °C, SD = 2.9) compared to those without health problems (26.2 °C, SD = 2.8). Seasonal analysis showed that mean LST was highest in spring (27.0 °C, SD = 3.7) and winter (27.1 °C, SD = 4.8), with individuals reporting health problems experiencing marginally higher temperatures, particularly in spring (27.3 °C vs. 26.6 °C) and summer (26.0 °C vs. 25.1 °C). The LST closest to the interview date was also higher among participants with health issues (30.8 °C, SD = 7.0) compared to those without (29.7 °C, SD = 7.9). The largest range of temperatures was observed for the LST closest to the interview date (7.8–38.5 °C), followed by the range during summer (14.7–35.3 °C) and winter (14.7–35.1 °C). These findings suggest a potential relationship between higher land surface temperatures and the presence of health problems.

**Table 2:** Descriptive statistics of environmental variables

	Total mean (SD)	Range (min - max)	No health problem mean (SD)	Some health problem mean (SD)
Mean LST (°C)	26.4 (2.8)	20.2–33.0	26.2 (2.8)	26.5 (2.9)
Mean Spring LST (°C)	27.0 (3.7)	19.9–36.3	26.6 (3.4)	27.3 (3.8)
Mean Summer LST (°C)	25.7 (5.8)	14.7–35.3	25.1 (5.6)	26.0 (5.9)
Mean Autumn LST (°C)	25.8 (2.7)	18.5–33.8	25.8 (2.9)	25.7 (2.6)
Mean Winter LST (°C)	27.1 (4.8)	14.7–35.1	27.3 (4.9)	27.0 (4.7)
LST closest to interview date (°C)	30.36 (7.35)	7.8–38.5	29.7 (7.9)	30.8 (7.0)

### EQ-5D-5 L dimension and severity level responses

**Fig. 1:**
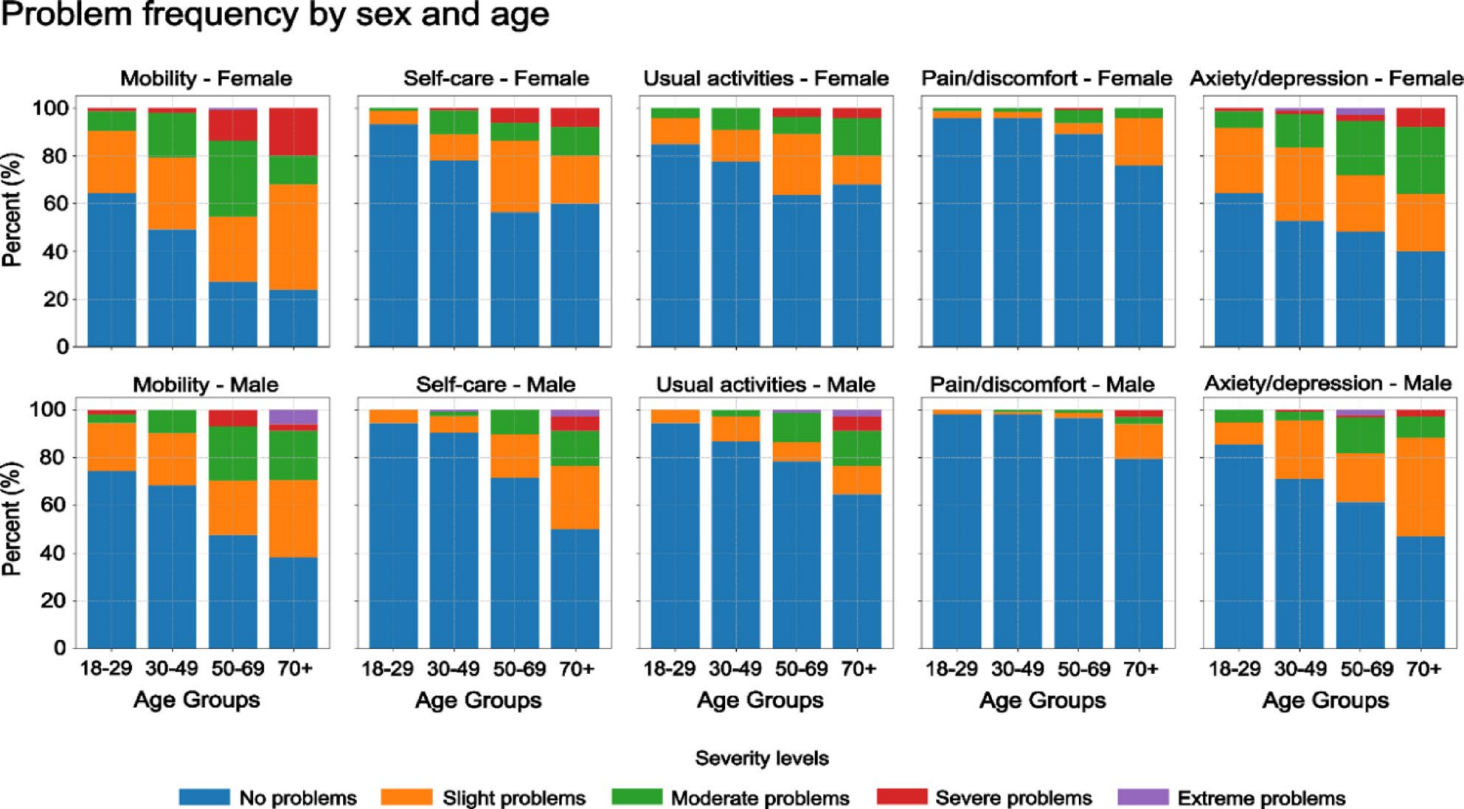
Fractions of responses across the five severity levels for each EQ-5D-5 L dimension, segmented by age group and sex

Figure 1 and Table S2 present the frequency of responses across the five severity levels for each EQ-5D-5L dimension, segmented by age group and sex. Mobility and anxiety or depression were the dimensions with the highest number of reported problems, affecting 49.1% and 31.9% of the participants, respectively. These issues were particularly prevalent among middle-aged and older women.

Generally, the frequency of reported health problems increased with age across all dimensions. Among the participants aged 50–69, moderate to severe mobility issues were reported by a notable proportion, while the youngest age group (18–29) showed strongly lower levels of reported problems. Across all dimensions, women reported HRQOL problems more frequently than men, indicating possible gender disparities in perceived quality of life.

### Associations and EQ-5D-5L dimensions

Table 3 presents an extract (for the full results consult SM_BEOI file in the supplements) of the results of analyses examining the association between short-term and long-term LST exposure and the EQ-5D-5L utility index; the results are reported with OR and 95% confidence intervals. The first row presents the analysis for the beta regression on values 0 < utility index < 1. The next rows present the results for the dichotomous utility index (utility index = 1, perfect health). For the continuous utility index, no significant association with LST exposure was detected among the participants (*n* = 440) who did not report perfect health (0 < utility index < 1).

However, when considering the binary outcome of reporting perfect health, utility index = 1 (*n* = 260) versus utility index < 1 (*n* = 440), significant associations emerged based on spatial variations in seasonal LST within the city. Residing in a location with a 1 °C higher mean spring LST compared to cooler locations was associated with 7% lower odds of reporting perfect health (OR = 0.93; 95% CI: 0.89–0.98). A similar pattern was observed for summer, where a 1 °C higher mean LST, relative to other intra-city locations, corresponded to 4% lower odds of reporting perfect health (OR = 0.96; 95% CI: 0.93–0.99). No such association was observed for LST during autumn, winter, or the temperature closer to the interview date.

Spring LST was associated with worse self-reported health dimensions. A 1 °C increase in spring LST with respect to cooler locations was associated with higher odds of reporting problems in usual activities (OR = 1.06; 95% CI: 1.01–1.12). Similarly, an association was observed for summer temperatures and anxiety or depression (OR = 1.04; 95% CI: 1.01–1.07). No associations were found for mobility, self-care or pain or discomfort across any LST metrics. Table S3 in supplementary materials present the Cox and Snell pseudo-R2 for the association’s analysis.

**Table 3:** Adjusted odd ratios (95% CI) in risk of reporting worst health (0 < utility index < 1, beta regression in blue) and reporting perfect health (Dichotomous utility index in blue) and per dimension (Logistic regression in orange) for the short- and long-term association with LST

	Mean LST (°C)	Mean Spring LST (°C)	Mean Summer LST (°C)	Mean Autumn LST (°C)	Mean Winter LST (°C)	LST closer to interview date (°C)
**0 < utility** **index < 1**	1.00 (0.98–1.02)	1.00 (0.98–1.02)	1.00 (0.99–1.01)	1.00 (0.97–1.03)	1.00 (0.98–1.01)	1.00 (0.99–1.01)
**Perfect health utility index = 1**	0.94 (0.88–1.00.88.00)	0.93 (0.89–0.98)	0.96 (0.93–0.99)	0.98 (0.92–1.05)	1.02 (0.98–1.05)	0.98 (0.96–1.00.96.00)
**Mobility**	1.01 (0.95–1.07)	1.01 (0.97–1.06)	1.02 (0.99–1.05)	0.99 (0.93–1.05)	0.98 (0.95–1.02)	1.01 (0.98–1.03)
**Self-care**	1.06 (0.99–1.14)	1.05 (1.00–1.11.00.11)	1.01 (0.98–1.05)	1.03 (0.96–1.12)	1.03 (0.98–1.07)	1.01 (0.99–1.04)
**Usual** **activities**	1.07 (0.99–1.15)	1.06 (1.01–1.12)	1.01(0.98–1.05)	1.03 (0.96–1.12)	1.02 (0.98–1.07)	1.02 (0.99–1.05)
**Pain or** **discomfort**	1.00 (0.90–1.12)	1.02 (0.93–1.11)	0.97 (0.92–1.03)	0.97(0.87–1.08)	1.05 (0.97–1.13)	0.97 (0.93–1.00.93.00)
**Anxiety or depression**	1.05 (0.99–1.11)	1.04 (1.00–1.09.00.09)	1.04 (1.01–1.07)	1.03 (0.97–1.09)	0.97 (0.94–1.01)	1.02 (0.99–1.04)

**Fig. 2:**
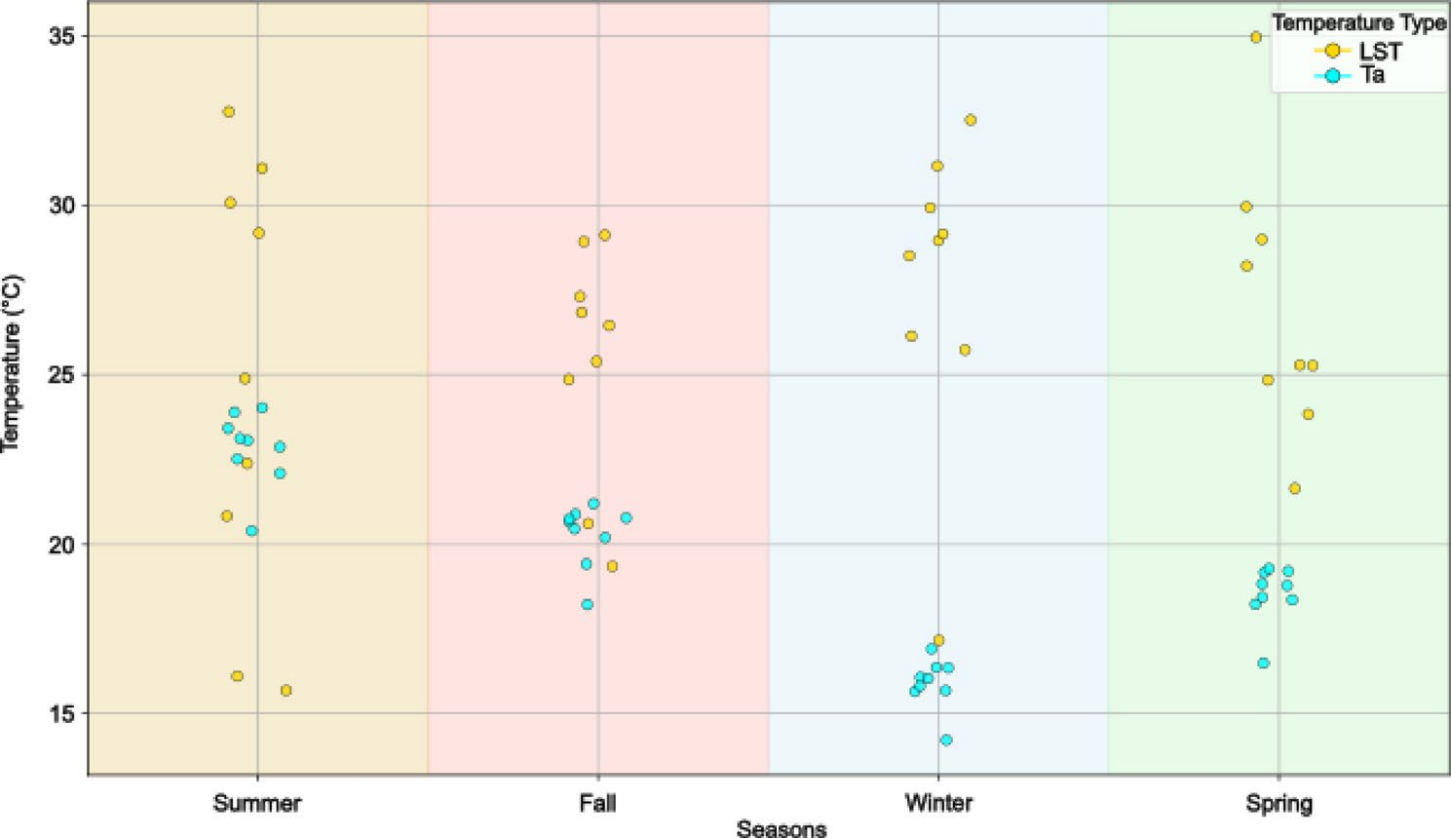
Comparison of land surface temperature and air temperature by season

Figure 2 illustrates the seasonal differences between Ta and LST. LST consistently exceeded Ta, with the largest differences observed during winter and spring. There was notable spatial variability in thermal behaviour across the houses. For instance, in summer, LST values for houses were widely dispersed across a range of temperatures, while Ta showed less spatial variation. This result suggests that in warmer seasons, LST is a better proxy for Ta, than during colder seasons. In addition, LST seems to capture spatial heterogeneity more effectively than ambient air temperature, particularly during warmer months.

### Sensitivity analyses and model performance

Model performance was assessed using pseudo-R² statistics, as detailed in Table S3. The BEOI models demonstrated adequate fit, with pseudo-R² values ranging from 0.26 to 0.27 across temperature exposure metrics. Logistic regressions assessing the individual dimensions from EQ-5D-5 L had a pseudo-R^2^ ranging from 0.10 to 0.20.

Stratified analyses by sex and income produced results consistent with the main models. Associations between higher LST and reported problems in usual activities and anxiety or depression remained in the same direction, although statistical significance was attenuated due to wider confidence intervals. Notably, a stronger association was observed for the self-care dimension among women, with higher mean LST (AOR = 1.11, 95% CI 1.02–1.22) and spring LST (AOR = 1.09, 95% CI 1.02–1.17) showing increased odds of reporting problems. These findings are consistent with the main results in both direction and magnitude, but statistical significance was reached only in this subgroup. Similarly, the sensitivity analysis for the lower-income group revealed comparable associations for usual activities and anxiety or depression and a stronger association for self-care, mirroring the pattern observed among women. The null association for the mobility and pain or discomfort dimensions were also robust to the stratification analysis.

The stratified analysis of the BEOI regression yielded AORs with similar directions and magnitudes across subgroups. Among females, the confidence interval widened, reducing the statistical significance of the association. In the low-income subgroup, the correlation maintained a comparable strength, with consistent magnitude and confidence interval estimates.

Interaction terms between temperature exposure and age or income were tested in the full models; none reached statistical significance (all *p* > 0.1). This was equally the case, for logistic and BEOI regressions, suggesting no substantial effect modification. Overall, these sensitivity checks indicate that the observed associations between temperature exposure and HRQOL are unlikely to be artefacts of demographic subgroups or model specification.

## Discussion

The results from our study involving residents of Lima, Peru, revealed that two out of three participants reported HRQOL problems, with an average EQ-5D-5L utility score of 0.91 (SD = 0.1). This score was lower than the population norms in Colombia [15] (mean = 0.953, SE = 0.001) and Belize [15] (mean = 0.947, SD = 0.002), which may be due to differences in sample representativeness. Our study focuses on a single city, while the comparative norms are countrywide.

We observed no association between LST and the utility index among participants who did not report perfect health (utility index between 0 and 1), across any of the temporal resolutions explored. This finding is consistent with a previous study conducted in southern Germany, where the authors also found no short-term association between air temperature and the EQ-5D-5 L utility index [17]. But contradicts a report from China where the authors observed that higher temperatures and fluctuations decreased self-report health scores [52]. The scarce evidence on the association between temperature and self-reported health is not conclusive. Nevertheless, there is evidence about how extreme temperatures can negatively affect other health outcomes, especially when combined with urban heat dynamics [5, 53, 54].

We also found evidence of an inverse association between spatial variation in spring and summer LST and the likelihood of reporting perfect health (utility index = 1). Additionally, higher spring and summer LST were associated with increased odds of reporting problems in the EQ-5D-5L dimensions of usual activities and anxiety or depression, respectively. The effect sizes in both analyses were small (1.0 < OR < 1.1 in this context) and of similar magnitude, suggesting that limitations reported by participants not in perfect health were concentrated in these two dimensions.

An association with the usual activities dimension of the EQ-5D-5L was also reported by Sohail et al., who examined short-term air temperature as the exposure [17]. In their study, the observed effect size was larger when comparing several degrees of difference. The authors hypothesized, based on previous literature, that the physiological stress caused by extreme temperatures could be driving this association [55, 56].

The association between temperature and anxiety or depression we observed, supports a large body of literature connecting non-optimal temperatures with mental health challenges. Rising temperatures may contribute to stress, fatigue, and poor-quality sleep, exacerbating anxiety or depressive symptoms [57]. Studies from various contexts have observed similar trends, where ambient temperature increases correlate with mental health decline [22, 58].

Although the associations between land surface temperature (LST) and HRQOL indicators appear small at the individual level, even modest reductions in HRQOL could have meaningful implications in a city like Lima, with more than 10 million of urban dwellers [39]. Small declines in HRQOL, if experienced broadly, may contribute to increased healthcare demand, reduced productivity, and a greater burden of symptoms such as anxiety. These cumulative effects could exacerbate in Lima, where almost one of every four inhabitants resides in informal settlements with limited capacity to adapt to heat exposure [40].

Interestingly, we did not observe a significant temperature association on the mobility dimension, which contrasts with some studies linking high temperatures with reduced physical activity [59–61]. This discrepancy could result from differences in climate adaptability or activity types among populations. Similarly, no significant association between temperature and pain or discomfort was observed, possibly due to individual variability in pain sensitivity and perception, confounded by other factors such as pre-existing health conditions [62]. The association between temperature and pain is inhomogeneous in the literature [63–65]. No association was also observed on the self-care dimension. Although research directly linking temperature to self-care was not found, warmer environments have been shown to affect productivity [66, 67] and cognitive focus [68].

The lack of statistically significant associations above the described led to some consideration. The minimal differences in mean LST between participants reporting no versus some health problems (typically < 1 °C) indicate limited exposure contrast, which likely reduced the statistical power to detect associations. Furthermore, the EQ-5D-5L instrument is well-known for its ceiling effects, as the majority of respondents reported no health problems in domains such as pain or discomfort and self-care, thereby constraining the variability in outcome measures. It is also plausible that the EQ-5D-5L is insufficiently sensitive to capture acute or moderate environmental stressors such as heat exposure. Finally, behavioural adaptations, physiological acclimatization, and social resilience within these communities, not considered in the experimental design due to its exploratory nature, may have buffer the perceived or actual impacts of elevated ambient temperatures, contributing to the observed null results. Although LST exposures occurred prior to survey administration, ensuring that exposure preceded outcome, the absence of clearly defined short- and long-term exposure windows may also have limited the experiment’s ability to detect associations between LST or air temperature and HRQOL.

The comparative analysis of LST and Ta in Fig. 2 highlights seasonal and station-specific differences between surface and air temperatures. Notably, the larger divergences were observed during winter and spring, likely influenced by spatio-temporal factors and microclimatic effects, such as the urban heat island effect [26]. Naserikia et al. reported that LST consistently exceeds Ta in Sydney, Australia, with the greatest difference in summer and the smallest in winter [69]. Similarly, we found that LST was generally higher than Ta, although the largest difference occurred in winter. This winter disparity was due to elevated LST values, while the Ta remained low, as expected for the season.

The high winter LST values may stem from the timing of data capture by Landsat 8, which passes over Lima around 10:10 am local time. At this time of the day, the LST is often about half of its daily maximum absolute value. While Ta measurements were collected at varying times, LST is consistently captured at the same time each day. Additionally, the emissivity of materials around houses and cloud patterns may influence the LST values.

The LST-Ta differences were smaller for the summer and spring compared to the winter, and these were the seasons where we observed a potential association of LST on outcomes. However, in fall, the LST-Ta difference was smaller than in spring, and we did not observe an association between LST and the odds of reporting HRQOL issues in this season. These observations could indicate a complex relationship between HRQOL and Ta-LST. For future studies using similar methodologies, we recommend presenting a comparison between the proxy variable LST and the target variable Ta, as illustrated in Fig. 2, which helps clarify the relationship between the two.

The sensitivity analyses indicated that the results were generally robust across both subgroup analyses and model specifications. However, the attenuation of some associations among females, contrasted with the strengthening of associations within the low-income subgroup, suggests that income and its implications for material conditions and health, may play a more influential role than sex differences. An alternative explanation could be the impact of sample size on confidence interval variability, as the female subgroup (*n* = 409) was smaller than the low-income subgroup (*n* = 599).

To our knowledge, this is the first study using remote sensing-derived temperature measurements to assess the potential association with HRQOL in a developing urban context, demonstrating a cost-effective method for regions with limited environmental monitoring infrastructure. Standardised questionnaires ensured reliable data collection across socioeconomic, behavioural and environmental factors.

### Strengths and limitations

This is one of the first works investigating the potential effect of LST on HRQOL. The methodology included the use of freely available satellite data as exposure variables, which made it suitable for reproducibility in other contexts with the same or different outcome variables. The high spatial resolution from the Landsat 8 TIRS instrument allows for exploring the differences in LST within the same city, while normally variations of temperature in time are more studied. Detecting differences in temperature within a city is difficult using air temperature due to most cities lacking a dense ground station network. The standardised questionnaires supported the reproducibility and diminished the bias in the data. We aimed for a Global South setup where more research about the influence of environmental exposures on populations is required.

These results must be interpreted with an awareness of some limitations. The lack of high-resolution humidity data is a notable gap. Humidity influences heat perception and could interact with temperature association on HRQOL. Additionally, the reliance on self-reported measures introduces potential recall and social desirability biases. While our cross-sectional design limits the ability to establish temporality, our analytical approach aimed to approximate a causal effect of temperature on HRQOL by adjusting for key confounders. Causal interpretation remains contingent on the validity of these assumptions, particularly regarding confounding control, exposure and outcome timing.

The LST mean, representing the decade before the health data collection, indicates that while the residential location in Lima is constant, some participants might have changed their address during this period, thereby not experiencing the exposure as originally recorded. Since participant mobility was only assessed over a one-year period, this could lead to exposure misclassification and potentially underestimate the observed associations. LST values extracted within a 30 m radius around each household, at a fixed time, may not fully capture individual thermal exposure, as indoor temperatures can differ due to building materials, ventilation, and microclimatic conditions and temporal variations throughout the day. Nonetheless, LST offers a consistent and spatially detailed proxy for indoor thermal exposure, where direct indoor measurements are not feasible, for large-scale comparisons.

## Conclusion and recommendations

Our study reveals that higher temperatures in Lima, Peru, are associated with lower health-related quality of life , particularly affecting the likelihood of reporting perfect health and increasing problems in usual activities and anxiety or depression as measured by the EQ-5D-5L. These associations were most evident during spring and summer, highlighting residents’ vulnerability to seasonal temperature variations.

By applying a methodologically innovative approach, this research advances environmental epidemiology in developing urban contexts and offers a replicable framework for assessing temperature, health relationships where high-resolution environmental data are scarce. The findings emphasize the urgent need for further investigation into how environmental exposures, especially rising temperatures, influence population health, an issue of growing importance under climate change.

Future studies should explore how heat disproportionately affects vulnerable groups, such as older adults and individuals with chronic conditions, and strengthen evidence through larger sample sizes. Incorporating the thermal dimension into urban planning as well as urban interventions in unplanned communities, through tree planting, green space expansion, improved building materials, and community-based cooling initiatives, can help protect public health in rapidly warming cities of the Global South.

## Supplementary Information

Supplementary Material 1.

Supplementary Material 2.

Supplementary Material 3.

Supplementary Material 4.

## Data Availability

Data supporting the results of this study can be obtained from the corresponding author upon reasonable request.
